# Prediction and Prevention of Preterm Birth: Secondary Analysis of a Randomized Intervention Trial

**DOI:** 10.3390/jcm12175459

**Published:** 2023-08-23

**Authors:** C. Andrew Combs, John A. F. Zupancic, Michael Walker, Jing Shi

**Affiliations:** 1 Pediatrix Center for Research, Education, Quality & Safety, Sunrise, FL 33323, USA; 2 Department of Neonatology, Beth Israel Deaconess Medical Center, Boston, MA 02215, USA; 3 Department of Pediatrics, Harvard Medical School, Boston, MA 02115, USA; 4 Statistics Consultant, Carlsbad, CA 92009, USA

**Keywords:** 17-hydroxyprogesterone caproate, care management, length of stay, low-dose aspirin, neonatal respiratory morbidity, preterm birth, proteomic biomarkers, risk assessment

## Abstract

Our objective was to evaluate whether pregnancy is prolonged by the use of a proteomics-based maternal serum screening test followed by treatment interventions. This is a secondary analysis of the PREVENT-PTB randomized trial comparing screening with the PreTRM test versus no screening. The primary trial analysis found no significant between-group difference in the preterm birth rate. Rather than considering a dichotomous outcome (preterm versus term), we treated gestational age at birth as a continuous variable using survival analysis. We also evaluated between-group difference in NICU length of stay and duration of respiratory support. Results indicated that pregnancy was significantly prolonged in subjects screened with the PreTRM test compared to controls (adjusted hazard ratio 0.53, 95% confidence interval 0.36–0.78, *p* < 0.01). Newborns of screened subjects had significantly shorter NICU stays but no significant decrease in duration of respiratory support. In the PreTRM screen-positive group, interventions that were associated with pregnancy prolongation included care management and low-dose aspirin but not 17-hydroxyprogesterone caproate. We conclude that screening with the PreTRM test followed by interventions for screen-positive pregnancies may prolong pregnancy and reduce NICU LOS, but these observations need to be confirmed by additional research.

## 1. Introduction

Preterm birth (PTB) complicates over 10% of pregnancies in the USA [1] and is a leading cause of neonatal morbidity and mortality [2]. A potential strategy to reduce the rate of PTB is to identify patients at increased risk and to target specific interventions to those patients. As examples, vaginal progesterone reduces early PTB in patients with mid-trimester sonographic short cervix [3,4], and low-dose aspirin reduces preterm preeclampsia in patients with preeclampsia risk factors [5] and reduces spontaneous PTB in patients with prior PTB [6]. These interventions have been in widespread use for a decade, but the overall rate of PTB has not decreased, in part because only a small percentage of patients are identified as candidates for treatment.

A newer method of identifying patients at risk for PTB is the PreTRM™ test (Sera Prognostics Inc., Salt Lake City, UT, USA), developed through analysis of the maternal serum proteome. This test designates a patient at increased risk if a second-trimester blood sample has an elevated ratio of insulin-like growth factor binding protein-4 (IGFBP4) to sex hormone binding globulin (SHBG) [7]. The test is a significant predictor of both indicated and spontaneous PTB < 32 weeks, neonatal morbidity, and neonatal length of stay (LOS) [8,9], and has been suggested to be both cost-effective and cost-saving [10,11].

Prediction of PTB is of clinical value only if it leads to interventions that reduce the risk of PTB or its complications. For patients identified by the PreTRM test as having increased PTB risk, Branch et al. [12] hypothesized that PTB could be reduced by a suite of interventions including progestogen treatment, low-dose aspirin, and a care management protocol comprising increased outreach, patient education, and specialist care. To test this, they conducted the PREVENT-PTB trial, in which patients without traditional PTB risk factors were randomly allocated to be screened with the PreTRM test versus no screening. Screened patients who were identified as high-risk by the test were offered the interventions. The results showed no significant between-group difference in the median gestational age at birth (GA_birth_) or in the proportion with PTB < 37 wks. However, early termination of the trial due to funding restrictions left it underpowered for these outcomes. Interestingly, newborns in the screened group had shorter LOS in the neonatal intensive care unit (NICU) following PTB and a trend toward improved neonatal morbidity. The authors speculated that these improvements may have resulted from a lower rate of PTB < 35 weeks in the screened group but did not evaluate this further.

The primary analysis of the PREVENT-PTB trial appropriately followed a prespecified statistical analysis plan (SAP). However, there is substantial loss of information when a continuous outcome such as GA_birth_ is dichotomized using arbitrary cut-points such as PTB < 37 weeks. Dichotomization might mask clinically relevant differences of several days or even weeks in early PTBs. Further, the median GA_birth_ in the PREVENT-PTB trial (39.1 weeks in both groups) was largely driven by the majority of patients who delivered at term. We hypothesized that analysis of GA_birth_ as a continuous variable with a focus on the decile of patients with the earliest births might reveal a significant difference that would explain the observed shorter LOS and trend toward reduced morbidity among those randomized to screening. The present study was designed to test this hypothesis. In addition, we also sought to explore which, if any, of the interventions was associated with increased GA_birth_.

## 2. Materials and Methods

### 2.1. Synopsis of the PREVENT-PTB Trial

The PREVENT-PTB trial has been previously described in detail [12]. Briefly, the study included patients without current or historical risk factors for PTB (current pregnancy with multifetal gestation, signs or symptoms of preterm labor, rupture of membranes, sonographic cervical length < 25 mm, major fetal structural or genetic anomalies, or medical conditions that increase PTB risk; history of prior PTB, uterine anomaly or cervical conization). Subjects were excluded if they used or planned to use heparin or low-dose aspirin during the pregnancy. Subjects were randomly allocated to have the PreTRM test between 19^5/7^ and 20^6/7^ weeks of gestation (screened group, N = 595) versus standard obstetric care without the PreTRM test (control group, N = 596). In the screened group, a PreTRM test result indicating ≥14% risk of PTB was considered screen-positive and occurred in 33% (196 of 595). Screen-positive patients were offered prophylactic progestogen (either 17-hydroxyprogesterone caproate (17OHPC) 250 mg weekly or vaginal progesterone 200 mg each night), low-dose aspirin (81 mg daily), sonographic cervical length measurement, and care management (visits to a high-risk clinic, weekly phone contact, a smartphone app for symptom review, and access to 24 h support). The trial was prospectively registered on clinicaltrials.gov (NCT 03530332), approved by Intermountain Healthcare’s institutional review board, and conducted with the informed consent of all participants.

The primary outcome was the rate of spontaneous PTB < 37 weeks. Using an adaptive study design, a planned sample size of 3000 to 10,000 patients was targeted to power the trial to detect a reduction in spontaneous PTB from 6.4% in controls to 4.7% in screened patients. The trial was terminated at <40% of the planned sample size owing to limited funding.

The primary outcome occurred in 2.7% of the screened group and in 3.5% of controls (*p* = 0.41). Rates of PTB < 35 weeks (0.2% versus 0.8%, respectively) and PTB < 32 weeks (0.2% versus 0.3%, respectively) were similar in the two groups. Median neonatal LOS was similar (1.9 days in both groups) overall, but NICU LOS was significantly shorter for preterm newborns in the screened group (7.6 days versus 36.7 days, *p* = 0.028), a prespecified secondary outcome not corrected for multiple comparisons. Fewer preterm newborns in the screened group had high scores on a composite morbidity and mortality index (16% versus 31%, *p* = 0.24) [13].

### 2.2. Secondary Analysis

For the secondary analysis, the clinician investigators (C.A.C., J.A.F.Z.) met and agreed upon an SAP without having access to the primary data from the PREVENT-PTB trial. The trial sponsor, Sera Prognostics, Inc., released the requested de-identified primary data to the statistician investigators (M.W., J.S.), who suggested minor modifications to the SAP after a preliminary review.

Our primary outcome of interest was GA_birth_ as a continuous outcome, comparing the screened group versus controls. To avoid diluting the outcome by the majority who delivered at term, we restricted the analyses to the subgroup defined by the earliest decile of each group, i.e., the 10% of subjects with the lowest GA_birth_. Although it might have seemed more intuitive to restrict the analysis to those with PTB < 37 weeks, we knew from the trial publication that the number of such births was too small to support our planned analyses. We generated Kaplan–Meier survival plots of GA_birth_ in each subgroup and calculated hazard ratios using Cox proportional hazards models with and without adjustment for maternal age (dichotomized as <40 years versus ≥40 years [14]) and parity (dichotomized as nulliparous versus parous [15,16]). We repeated all these analyses after excluding the subgroup of patients who were treated with 17OHPC because the U. S. Food and Drug Administration has withdrawn approval of this medication [17], and we wished to determine whether absence of 17OHPC treatment would influence the result.

Analysis of NICU LOS was similarly restricted to the earliest decile of each group. This analysis was also restricted to those who were admitted to NICU because NICU LOS is technically not defined for patients not admitted to NICU. Cox models for NICU LOS included hazard ratios with and without adjustment for maternal age, parity, and GA_birth_.

Neonatal intensive respiratory support was defined as ventilator use (with intubation), high flow nasal cannula (≥2 L/min), continuous positive airway pressure, or nasal intermittent mechanical ventilation. Analysis of respiratory support was similarly restricted to the earliest decile and those who received any support. Cox models for respiratory support included hazard ratios with and without adjustment for maternal age, parity, and GA_birth_.

To evaluate which, if any, of the interventions contributed to pregnancy prolongation, we evaluated the GA_birth_ outcome in the screen-positive patients in the screened group, comparing those who declined all the offered interventions to those who accepted some or all of the interventions, in various combinations. The specific interventions analyzed were progestogens, low-dose aspirin, and care management. We generated Kaplan–Meier plots of GA_birth_ comparing intervention versus declined-intervention subgroups and calculated hazard ratios using Cox proportional hazards models with and without adjustment for maternal age and parity.

Analyses were performed using R software version 4.2.2 [18]. Between-group differences in hazard ratios were evaluated using the log-rank test, with 2-tailed *p*-values < 0.05 considered significant. No correction for multiple comparisons was made.

## 3. Results

From the total trial pool of 1191 randomized subjects, the earliest decile of GA_birth_ comprised 123 subjects (63 in the screened group, GA_birth_ ≤ 37^4/7^ weeks; 60 in controls, GA_birth_ ≤ 37^3/7^ weeks).

**Figure 1:**
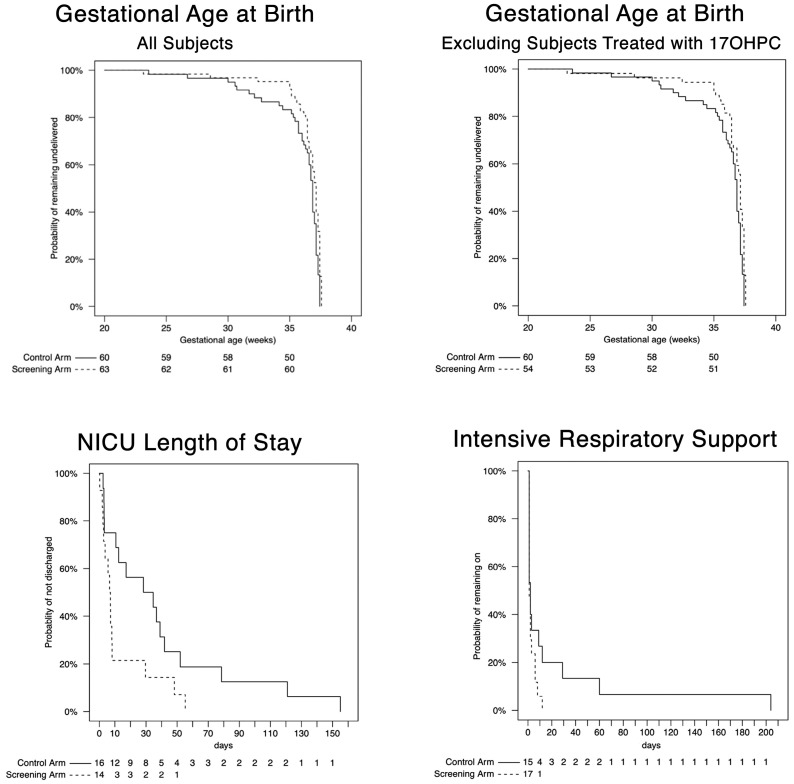
Kaplan–Meier plots for the lowest decile of gestational age at birth in subjects screened with the PreTRM test (dashed curves) versus subjects not screened (solid curves). Numbers below each graph are numbers of subjects in each group remaining at each time point. Upper panels: gestational age at birth for all subjects (**left**) and excluding 9 subjects treated with 17-hydroxyprogesterone caproate (17HOPC, **right**); difference significant at *p* < 0.005 for both, log-rank test. (**Lower left**): length of stay in neonatal intensive care unit (NICU), difference significant at *p* = 0.032. (**Lower right**): Days of intensive respiratory support, difference not significant (*p* = 0.15). In lower panels no included subjects were treated with 17OHPC.

The survival plots of GA_birth_, NICU LOS, and days of respiratory support in screened versus control subjects in the earliest decile are shown in Figure 1. For GA_birth_, there was a distinct separation between the curves between 32 weeks and 37 weeks reflecting fewer births in the screened group at these gestational ages, as shown in the upper left panel of Figure 1. This separation persisted after excluding the nine patients who were treated with 17OHPC in the earliest decile, as shown in the upper right panel. The adjusted hazard ratio 0.53 (95% CI, 0.36–0.78) was significant (*p* < 0.01, Table 1), indicating a prolongation of pregnancy in the screened group. This effect persisted after excluding those treated with 17OHPC. However, despite this prolongation, the median GA_birth_ was similar in the screened subjects (37.1 weeks; interquartile range (IQR), 36.4–37.4 weeks) compared to control subjects (36.9 weeks; IQR, 35.7–37.1). The prolongation of pregnancy in the screened group was associated with shorter NICU LOS, as shown in the middle panel of Figure 1 and reflected in the adjusted hazard ratio 2.84 (95% CI, 1.12–6.70). Although duration of respiratory support was not significantly different between the two groups, the lower right panel of Figure 1 is suggestive of a trend toward shorter duration in the screened group. After adjustment for GA_birth_, there was no longer a significant difference in NICU LOS or any trend toward shorter duration of respiratory support.

**Figure 2:**
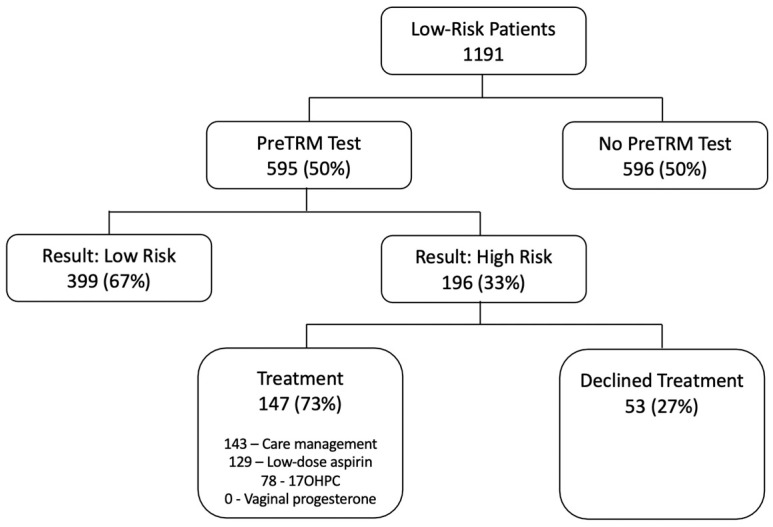
Flow chart summarizing number of subjects in each group and subgroup of the trial. The individual treatments sum to more than 147 because most patients had more than 1 treatment. Abbreviation: 17-OHPC = 17-hydroxyprogesterone caproate.

**Figure 3:**
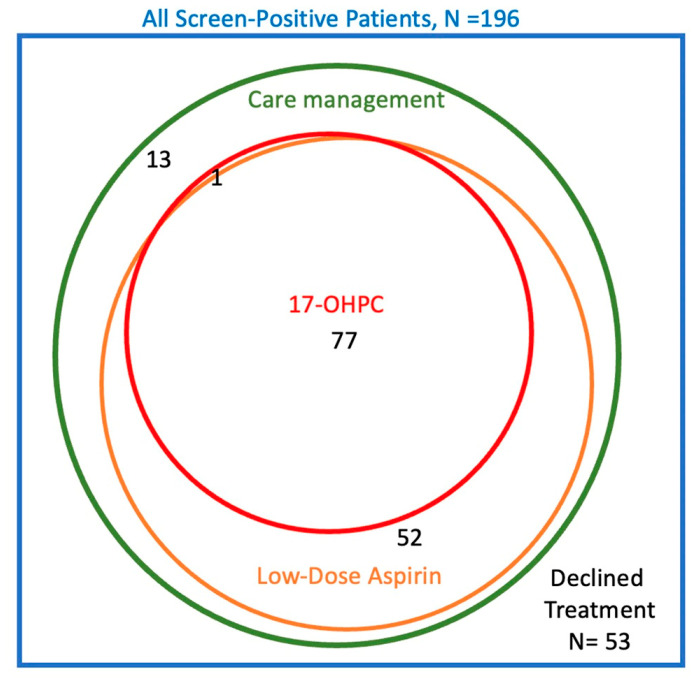
Venn diagram of treatments chosen by screen-positive subjects. The number in each region is the number of subjects. Abbreviation: 17-OHPC = 17-hydroxyprogesterone caproate.

The flow chart in Figure 2 summarizes the treatments chosen by the 196 subjects in the screened group who were screen-positive and the Venn diagram in Figure 3 shows the overlaps in treatments chosen. In total, 53 subjects (27%) declined all the offered interventions, 143 (73%) in total elected care management, 129 (66%) elected low-dose aspirin, 78 (40%) elected prophylactic 17OHPC, and none used prophylactic vaginal progesterone. There were large overlaps in the treatments chosen. All patients who used either aspirin or 17OHPC were also enrolled in care management; only 13 subjects had care management alone without either medication. Similarly, 77 of 78 subjects (99%) who elected 17OHPC also took aspirin. On the other hand, 52 of 129 subjects (40%) who elected aspirin did not use 17OHPC. Because of these overlaps, it was not possible to evaluate the independent association of each treatment with GA_birth_. Instead, we analyzed GA_birth_ among those who declined all treatment versus three comparison groups: those who accepted any-or-all treatments; those who had care management with-or-without aspirin but without 17OHPC; and those who had care management alone.

Table 2 summarizes the Cox regression statistics for these comparisons. There was no overall difference in GA_birth_ comparing those who had any treatment versus those who declined all treatments. Those who had care management with-or-without aspirin but who did not have 17OHPC versus those who declined treatment had significant prolongation of pregnancy (adjusted hazard ratio 0.66, 95% CI 0.46–0.97, *p* = 0.03). A similar prolongation was noted among those with care management alone compared to those who declined treatment (adjusted hazard ratio 0.48, 95% CI 0.24–0.94, *p* = 0.03).

## 4. Discussion

The principal finding of this secondary analysis of the PREVENT-PTB trial is that screening with the PreTRM test was associated with a significant prolongation of pregnancy compared to no screening among patients in the earliest decile of GA_birth_, a subset that cannot be identified a priori. The prolongation is detectable as a separation of the survival curves between 32 and 37 weeks of gestation with GA_birth_ analyzed as a continuous variable. The prolongation was not detected in the primary analysis of the trial, in which GA_birth_ was dichotomized (i.e., <37 versus ≥37 weeks of gestation, term versus preterm). The prolongation also was not reflected in an increase in median GA_birth_. These observations underscore the importance of analyzing continuous variables as continuous variables and avoiding the data loss that occurs when collapsing them into dichotomous or categorical variables or expressing them as a synopsis measure of central tendency, such as median or mean.

We suggest that the pregnancy prolongation in the group screened with the PreTRM test, while modest, is clinically relevant. Newborns of subjects who had screening with the PreTRM test had shorter NICU LOS. There was also a trend toward less respiratory morbidity in the screened group, though the statistical power was limited by the small number of subjects. Adjustment for GA_birth_ attenuated these effects (rightmost columns of Table 1), suggesting that these benefits of screening and treatment are likely entirely attributable to the reduction in early PTB in the screened group.

Because of the overlap in treatments chosen, we are unable to make definitive statements about whether the prolongation of pregnancy seen in the screened group was due to the use of care management, aspirin, or both in combination. It is reassuring that the benefits persisted after the exclusion of subjects treated with 17OHPC, now that this drug is no longer approved for use in the United States.

Care management alone may reduce PTB in patients at increased risk, as suggested by a recent review [19] that summarized several prior systematic reviews with mixed results and concluded that it might be “potentially beneficial” and cost-effective for patients at highest risk of PTB. Our results support the suggestion of efficacy, though only a small number of subjects in the PREVENT-PTB trial had care management alone without pharmacoprophylaxis. Care management can include diverse interventions such as patient navigation, high-risk clinic visits, nutrition and exercise counseling, smoking cessation assistance, doula services, mental health services, and financial counseling and coordination. Future research is needed to determine which components are beneficial and to evaluate cost effectiveness and potential harms.

Prophylactic low-dose aspirin has been associated with reduction of recurrent PTB in patients with prior PTB [6] and our results are suggestive of a potential benefit for patients selected by a positive PreTRM test. However, at this time, the American College of Obstetricians and Gynecologists does not endorse the use of aspirin for prevention of PTB in patients who lack preeclampsia risk factors [20]. Universal low-dose aspirin for preeclampsia prevention has been suggested by some, but concerns have been raised about the potential for maternal and fetal/neonatal bleeding complications [21,22].

Strengths of the study include the prospective randomized design of the parent trial. A strength of the secondary analysis is the use of statistical methods that assess GA_birth_ as a continuous outcome rather than a dichotomous variable.

There are also several limitations. First, this is a post hoc, secondary analysis conducted after the primary analysis was reported. Second, the number of PTB cases was small because of the early termination of the trial. Third, we did not have data on the use of antenatal corticosteroids, which can impact preterm neonatal respiratory morbidity. Fourth, the specific treatments chosen in response to the PreTRM test were based on patient preferences, not randomization, and thus the analysis of treatments reveals only associations and not necessarily a causal link between treatments and outcomes. Finally, we did not employ any statistical corrections to adjust for the large number of hypotheses tested; thus, the probability of Type 1 statistical error is larger than the nominal 5%.

Given the limitations, we consider our results to be exploratory only, suggesting potential topics for future trials. We suggest that there is a need for large, prospective trials to evaluate the efficacy of specific interventions such as care management and low-dose aspirin in patients identified at increased risk for PTB by the PreTRM test. Ongoing trials may provide additional insights [23,24].

## 5. Conclusions

Screening with the PreTRM test in the PREVENT-PTB trial appears to be associated with significant prolongation of pregnancy and this prolongation appears to be accompanied by improvement in neonatal outcome as reflected by shorter NICU LOS. Because this is a post hoc analysis, future research is needed to confirm these observations and to evaluate the individual contributions of care management, low-dose aspirin, and perhaps other interventions in achieving these apparent benefits.

## Figures and Tables

**Table 1:** Cox proportional hazards models among subjects in the earliest decile of gestational age at birth.

Comparison	Unadjusted Hazard Ratio (95% CI)	*p*-Value	Adjusted ^a^ Hazard Ratio (95% CI)	*p*-Value	Adjusted ^b^ Hazard Ratio (95% CI)	*p*-Value
GA_birth_, screened versus control, all subjects	0.56 (0.38–0.81)	<0.01	0.53 (0.36–0.78)	<0.01	--	--
GA_birth_, screened versus control, excluding those treated with 17OHPC	0.54 (0.36–0.81)	<0.01	0.51 (0.34–0.76)	<0.01	--	--
NICU length of stay, screened versus control, all subjects ^c^	2.28 (1.05–4.94)	0.03	2.84 (1.21–6.70)	0.02	1.44 (0.61–3.38)	0.41
Respiratory support, screened versus control, all subjects ^c^	1.74 (0.81–3.74)	0.15	1.82 (0.84–3.94)	0.13	0.61 (0.24–1.36)	0.31

Abbreviations: 17OHPC = 17-hydroxyprogesterone caproate. CI = confidence interval. GA_birth_ = gestational age at birth. NICU = neonatal intensive care unit. Hazard ratios <1 reflect longer duration in the screened group and ratios >1 reflect longer durations in the unscreened group. *p*-values from log-rank test. ^a^ Adjusted for maternal age (<40 versus ≥40 years) and parity (nulliparous versus parous). ^b^ Adjusted for maternal age, parity, and GA_birth_. ^c^ There were no subjects treated with 17OHPC in this subset.

**Table 2:** Cox proportional hazards models of associations between treatments and gestational age at birth in screen-positive subjects based on the PreTRM test.

Comparison	Number Treated	Number Declining Treatment	Unadjusted Hazard Ratio (95% CI)	*p*-Value	Adjusted ^a^ Hazard Ratio (95% CI)	*p*-Value
Any treatment versus declined treatment, all subjects	143	53	0.86 (0.62–1.18)	0.34	0.89 (0.65–1.23)	0.49
Any treatment versus declined treatment, excluding those treated with 17OHPC	65	53	0.65 (0.45–0.94)	0.02	0.66 (0.46–0.97)	0.03
Care management alone versus declined any treatment	13	53	0.48 (0.24–0.94)	0.03	0.47 (0.24–0.94)	0.03

Abbreviation: 17OHPC = 17-hydroxyprogesterone caproate. Hazard ratios <1 reflect longer duration in the treated group. *p*-values from log-rank test. ^a^ Adjusted for maternal age (<40 versus ≥40 years) and parity (nulliparous versus parous).

## Data Availability

We requested a relevant subset of the PREVENT-PTB trial data from Sera Prognostics, Inc., the trial sponsor, upon presentation of our statistical analysis plan. We are not authorized to share this proprietary, privately held data. Investigators with reasonable requests are encouraged to seek trial data directly from the sponsor.

## References

[1] Osterman M.J.K., Hamilton B.E., Martin J.A., Driscoll A.K., Valenzuela C.P. (2023). Births: Final data for 2021. Nat. Vital. Stat. Rep..

[2] Manuck T.A., Rice M.M., Bailit J.L., Grobman W.A., Reddy U.M., Wapner R.J., Thorp J.M., Caritis S.N., Prasad M., Tita A.T. (2016). Preterm neonatal morbidity and mortality by gestational age: A contemporary cohort. Am. J. Obstet. Gynecol..

[3] Romero R., Nicolaides K., Conde-Agudelo A., O’Brien J.M., Cetingoz E., Da Fonseca E., Creasy G.W., Hassan S.S. (2016). Vaginal progesterone decreases preterm birth ≤34 weeks of gestation in women with a singleton pregnancy and a short cervix: An updated meta-analysis including data from the OPPTIMUM study. Ultrasound Obstet. Gynecol..

[4] EPPPIC Group (2021). Evaluating progestogens for preventing preterm birth International Collaborative (EPPPIC): Meta-analysis of individual participant data from randomised controlled trials. Lancet.

[5] Henderson J.T., Vesco K.K., Senger C.A., Thomas R.G., Redmond N. (2021). Aspirin use to prevent preeclampsia and related morbidity and mortality. Updated evidence report and systematic review for the US Preventive Services Task Force. JAMA.

[6] Kupka E., Hesselman S., Hastie R., Lomartire R., Wikström A.K., Bergman L. (2023). Low-dose aspirin use in pregnancy and the risk of preterm birth: A Swedish register-based cohort study. Am. J. Obstet. Gynecol..

[7] Saade G.R., Boggess K.A., Sullivan S.A., Markenson G.R., Iams J.D., Coonrod D.V., Pereira L.M., Esplin M.S., Cousins L.M., Lam G.K. (2016). Development and validation of a spontaneous preterm delivery predictor in asymptomatic women. Am. J. Obstet. Gynecol..

[8] Markenson G.R., Saade G.R., Laurent L.C., Heyborne K.D., Coonrod D.V., Schoen C.N., Baxter J.K., Haas D.M., Longo S., Grobman W.A. (2020). Performance of a proteomic preterm delivery predictor in a large independent cohort. Am. J. Obstet. Gynecol..

[9] Burchard J., Polpitiya A.D., Fox A.C., Randolph T.L., Fleischer T.C., Dufford M.T., Garite T.J., Critchfield G.C., Boniface J.J., Saade G.R. (2021). Clinical validation of a proteomic biomarker threshold for increased risk of spontaneous preterm birth and associated clinical outcomes: A replication study. J. Clin. Med..

[10] Grabner M., Burchard J., Nguyen C., Chung H., Gangan N., Boniface J.J., Zupancic J.A., Stanek E. (2021). Cost-effectiveness of a proteomic test for preterm birth prediction. Clin. Outcomes Res..

[11] Burchard J., Markenson G.R., Saade G.R., Laurent L.C., Heyborne K.D., Coonrod D.V., Schoen C.N., Baxter J.K., Haas D.M., Longo S.A. (2022). Clinical and economic evaluation of a proteomic biomarker preterm birth risk predictor: Cost-effectiveness modeling of prenatal interventions applied to predicted higher-risk pregnancies within a large and diverse cohort. J. Med. Econ..

[12] Branch D.W., VanBuren J.M., Porter T.F., Holmgren C., Holubkov R., Page K., Burchard J., Lam G.K., Esplin M.S. (2023). Prediction and prevention of preterm birth: A prospective, randomized intervention trial. Am. J. Perinatol..

[13] Hassan S.S., Romero R., Vidyadhari D., Fusey S., Baxter J.K., Khandelwal M., Vijayaraghavan J., Trivedi Y., Soma-Pillay P., Sambarey P. (2011). Vaginal progesterone reduces the rate of preterm birth in women with a sonographic short cervix: A multicenter, randomized, double-blind, placebo-controlled trial. Ultrasound Obstet. Gynecol..

[14] Ferre C., Callaghan W., Olson C., Sharma A., Barfield W. (2016). Effects of maternal age and age-specific preterm birth rates on overall preterm birth rates—United States, 2007 and 2014. Morb. Mortal. Wkly. Rep..

[15] Shah P.S. (2010). Knowledge Synthesis Group on Determinants of LBW/PT births. Parity and low birth weight and preterm birth: A systematic review and meta-analysis. Acta Obstet. Gynecol. Scand..

[16] Koullali B., van Zijl M.D., Kazemier B.M., Oudijk M.A., Mol B.W.J., Pajkrt E., Ravelli A.C.J. (2020). The association between parity and spontaneous preterm birth: A population based study. BMC Pregnancy Childbirth.

[17] U.S. Food & Drug Administration FDA Commissioner and Chief Scientist Announce Decision to Withdraw Approval of Makena. https://www.fda.gov/news-events/press-announcements/fda-commissioner-and-chief-scientist-announce-decision-withdraw-approval-makena.

[18] Foundation R. The R Project for Statistical Computing. https://www.r-project.org.

[19] Garite T.J., Manuck T.A. (2023). Should case management be considered a component of obstetrical interventions for pregnancies at risk of preterm birth?. Am. J. Obstet. Gynecol..

[20] Committee on Obstetric Practice, Society for Maternal Fetal Medicine (2018). Low-dose aspirin use during pregnancy. ACOG Committee Opinion 743. Obstet. Gynecol..

[21] Wright D., Wright A., Tan M.Y., Nicolaides K.H. (2022). When to give aspirin to prevent preeclampsia: Application of Bayesian decision theory. Am. J. Obstet. Gynecol..

[22] Jiang Y., Chen Z., Chen Y., Wei L., Gao P., Zhang J., Zhou X., Zhu S., Zhang H., Du Y. (2023). Aspirin use during pregnancy may be a potential risk for postpartum hemorrhage and increased blood loss: A systematic review and meta-analysis. Am. J. Obstet. Gynecol..

[23] Hoffman M. Serum Assessment of Preterm Birth Outcomes Compared to Historical Controls: AVERT-PRETERM Trial. NCT03151330. NCT03151330.

[24] Iriye B. Prematurity Risk Assessment Combined with Clinical Interventions for Improving Neonatal Outcomes (PRIME). NCT 04301518. NCT04301518.

